# Association between serum uric acid levels and clinical outcomes in patients with acute kidney injury

**DOI:** 10.1080/0886022X.2023.2169617

**Published:** 2023-04-19

**Authors:** Xunliang Li, Jianping Sun, Quandong Bu, Bin Zhou, Lin Li, Xiaofei Man, Long Zhao, Yan Xu, Hong Luan

**Affiliations:** Department of Nephrology, The Affiliated Hospital of Qingdao University, Qingdao, China

**Keywords:** Acute kidney injury, serum uric acid, mortality, prognostic

## Abstract

**Background:**

The effects of serum uric acid (SUA) on clinical outcomes in patients with acute kidney injury (AKI) are unclear. The aim of this study was to investigate the association of SUA levels with clinical outcomes of AKI patients.

**Methods:**

The data of AKI patients hospitalized in the Affiliated Hospital of Qingdao University were retrospectively reviewed. Multivariable logistic regression was utilized to assess the association between SUA levels and the clinical outcomes of AKI patients. Receiver operating characteristic (ROC) analysis was applied to assess the predictive ability of SUA levels for in-hospital mortality in patients with AKI.

**Results:**

A total of 4,646 AKI patients were eligible for study inclusion. In multivariable analysis, after adjustment for various confounding factors in the fully adjusted model, a higher SUA level was found to be associated with increased in-hospital mortality of AKI patients with an odds ratio (OR) of 1.72 (95% CI, 1.21–2.33, *p* = 0.005) for the SUA level >5.1–6.9 mg/dl group and 2.75 (95% CI, 1.78–4.26, *p* < 0.001) for the SUA level >6.9 mg/dl group compared with the reference group (SUA ≤3.6 mg/dl). In the ROC analysis, the area under the curve (AUC) of SUA was 0.65 with a sensitivity of 51% and a specificity of 73%.

**Conclusions:**

An elevated SUA level is associated with an increased risk of in-hospital mortality in patients with AKI, and it appears to be an independent prognostic marker for these patients.

## Background

Acute kidney injury (AKI) is a common and severe syndrome associated with high morbidity and mortality [1]. AKI occurs in 21% of hospitalized patients and sometimes in more than 50% of patients in intensive care units; the mortality rate of AKI patients is four times higher than that of non-AKI patients [2–4]. In addition, survivors often fail to recover renal function and require long-term dialysis, which imposes a significant financial burden [5–7]. Recognition of the clinical features that have the greatest impact on mortality in patients with AKI can help to guide supportive care for those most likely to benefit. Considering the high incidence and poor prognosis of AKI, several researchers have sought to identify risk factors for mortality in AKI.

Uric acid (UA) is the final metabolite of purine compounds. Approximately 70% of the uric acid in the body is excreted through the kidneys [8]. The serum uric acid (SUA) level is mainly determined by the estimated glomerular filtration rate (eGFR) and purine metabolism rate [9]. A large number of epidemiologic studies have suggested that hyperuricemia is an independent risk factor for mortality, cardiovascular disease, and kidney disease in the general population [10–13]. Hyperuricemia is very prevalent among chronic kidney disease (CKD) patients because of the decrease in renal excretion of UA when the eGFR declines. In addition, studies have demonstrated that hyperuricemia is a risk factor for all-cause mortality and cardiovascular events in the earlier stages of CKD but not renal replacement therapy [14,15]. The association between SUA levels and mortality has been inconsistent among studies of patients with end-stage renal disease who are receiving hemodialysis and peritoneal dialysis. Interestingly, in most studies involving patients receiving hemodialysis, elevated SUA levels were associated with lower mortality, which is called an ‘inverse epidemiological phenomenon’ [16–21]. However, there have been few studies on the relationship between SUA levels and AKI. Two studies demonstrated that hyperuricemia was associated with an increased incidence of AKI and all-cause mortality among hospitalized patients [22,23]. However, few epidemiological studies have investigated the impact of SUA levels on the prognosis of hospitalized patients with AKI [23]. The aim of this study was to evaluate the association between SUA levels and clinical outcomes in hospitalized patients with AKI.

## Materials and methods

### Inclusion and exclusion criteria

This study enrolled patients who developed AKI within 48 h of hospitalization at the Affiliated Hospital of Qingdao University from January 2015 to July 2020. AKI patients were identified according to the Kidney Disease: Improving Global Outcomes (KDIGO) 2012 criteria, which are as follows [24]: (1) an increase in serum creatinine to more than 1.5-fold the baseline values within the prior 7 days; (2) *a* ≥ 0.3 mg/dl increase in serum creatinine within the past 48 h; or (3) urine output <0.5 mL/kg/h for 6 h or more. We excluded the urine output criteria because most inpatients lacked urine volume records. The first serum creatinine value measured after hospital admission was used as the baseline serum creatinine concentration [25].

Patients were excluded if they met one of the following characteristics: (1) younger than 18 years; (2) hospitalization for <24 h; or (3) fewer than two serum creatinine tests. After excluding patients with less than two serum creatinine tests, the average number of serum creatinine tests for all patients was 3, and 58.8% of patients had 2 serum creatinine tests (Table S1). For patients who were admitted to the hospital more than once, only the first stay was included.

**Table 1.** Baseline characteristics.

	Uric acid (mg/dl)				
Variables	≤3.6 (*n* = 1174)	>3.6–5.1 (*n* = 1127)	>5.1–6.9 (*n* = 1189)	>6.9 (*n* = 1156)	*p* Value
Age	56.8 ± 17.8	56.9 ± 18.7	59.0 ± 17.7	59.5 ± 17.9	<0.001
Sex, male, n (%)	458 (39.0)	551 (48.9)	697 (58.6)	785 (67.9)	<0.001
BMI (kg/m^2^)	23.6 ± 3.4	24.4 ± 3.7	24.8 ± 3.9	25.1 ± 4.3	<0.001
Emergency status, n (%)	630 (53.7)	554 (49.2)	498 (41.9)	474 (41.0)	<0.001
AKI stage, n (%)					<0.001
STAGE 1	1028 (87.6)	1011 (89.7)	1088 (91.5)	1042 (90.1)	
STAGE 2	107 (9.1)	81 (7.2)	54 (4.5)	37 (3.2)	
STAGE 3	39 (3.3)	35 (3.1)	47 (4.0)	77 (6.7)	
Comorbidities, n (%)					
CKD	46 (3.9)	143 (12.7)	230 (19.3)	390 (33.7)	<0.001
Diabetes	226 (19.3)	294 (26.1)	333 (28.0)	298 (25.8)	<0.001
Hypertension	347 (29.6)	507 (45.0)	711 (59.8)	751 (65.0)	<0.001
Heart disease	171 (14.6)	285 (25.3)	402 (33.8)	415 (35.9)	<0.001
Cancer	265 (22.6)	221 (19.6)	198 (16.7)	216 (18.7)	<0.001
Biochemical indices					
Creatinine (μmol/L)^a^	110 ± 147	186 ± 259	262 ± 320	425 ± 404	<0.001
Albumin (g/L)^a^	37.3 ± 10.5	39.9 ± 10.4	41.2 ± 10.8	40.2 ± 10.9	<0.001
Cholesterol (g/L)^a^	3.85 ± 1.41	4.19 ± 1.46	4.33 ± 1.44	4.18 ± 1.47	<0.001
Triglyceride (g/L)^a^	1.11 ± 0.75	1.45 ± 1.10	1.69 ± 1.30	1.82 ± 1.34	<0.001
Hb (g/L)^a^	114 ± 26	117 ± 26	119 ± 27	114 ± 30	<0.001
Medication					
ACEI (%)	22 (1.9)	44 (3.9)	78 (6.6)	88 (7.6)	<0.001
ARB (%)	126 (10.7)	209 (18.5)	336 (28.3)	291 (25.2)	<0.001
BB (%)	452 (38.5)	505 (44.8)	659 (55.4)	666 (57.6)	<0.001
CCB (%)	227 (19.3)	304 (27.0)	451 (37.9)	474 (41.0)	<0.001
Furosemide (%)	192 (16.4)	232 (20.6)	290 (24.4)	424 (36.7)	<0.001
UA-lowering agent (%)	6 (0.5)	9 (0.8)	19 (1.6)	99 (8.6)	<0.001
eGFR, ml/min/1.73 m^2^	75.8 ± 25.5	61.3 ± 29.0	48.1 ± 28.2	29.8 ± 24.3	<0.001
RRT requirement	82 (7.0)	126 (11.2)	173 (14.6)	308 (26.6)	<0.001

BMI: body mass index; AKI: acute kidney injury; CKD: chronic kidney disease; Hb: hemoglobin; ACEI: angiotensin-converting enzyme inhibitor; ARB: angiotensin II receptor blocker; BB: beta blocker; CCB: calcium channel blocker; UA: uric acid; eGFR: estimated glomerular filtration rate; RRT: renal replacement therapy.

^a^The first values during the first day after admission were recorded.

### Data extraction

Data extracted from the electronic medical records included age, sex, body mass index (BMI), emergency status, AKI stage, and the presence of CKD, diabetes, hypertension, heart disease and cancer. Blood laboratory data, including SUA, creatinine, albumin (ALB), cholesterol, triglyceride and hemoglobin (Hb), were measured at the time of admission. Medications such as angiotensin-converting enzyme inhibitors (ACEIs), angiotensin receptor blockers (ARBs), beta receptor blockers, calcium channel blockers (CCBs), furosemide and UA-lowering agents were investigated. All biochemical indexes used the first value on the first day after admission. Hyperuricemia was defined as fasting blood uric acid levels higher than 7.0 mg/dl on two different days with a normal purine diet. AKI stages were defined as the maximum AKI stage within 48 h after hospital admission, which was determined by the creatinine level during the first 48 h after hospital admission. BMI was calculated as body weight divided by height squared (kg/m^2^). UA-lowering agent use was defined as the use of any UA-lowering agent, including febuxostat, allopurinol, and benzbromarone, after admission. The baseline eGFR was calculated using the CKD-EPI equation: eGFR = 141 × min (Scr/κ, 1)^α^ × max (Scr/κ, 1)^−1.209^ × 0.993^Age^ × 1.018 [if female]_1.159 [if black], where Scr is serum creatinine, κ is 0.7 for females and 0.9 for males, α is −0.329 for females and −0.411 for males, min indicates the minimum value of Scr/κ or 1, and max indicates the maximum value of Scr/κ or 1.

### Endpoints

The primary endpoint of this study was in-hospital mortality. Recovery of renal function was considered a secondary outcome and defined as discharge from the hospital with a creatinine level less than 1.5 times the baseline value.

### Management of missing data

Missing data variables are common in databases. In this study, missing values for all variables accounted for less than 5% of all data. The missing values were replaced by mean or median values.

### Statistical analysis

Continuous variables are expressed as medians [interquartile ranges (IQRs)] or means ± standard deviations (SDs) in the present study and were compared using analysis of variance. Categorical variables are expressed as numbers and percentages. The chi-square test or Fisher’s exact test was used as appropriate. The Kaplan–Meier method and log-rank tests were used to compare survival distributions among patients grouped according to SUA quartiles. According to previous studies, SUA and prognosis are not linearly related [9,22], so the SUA levels in this study were classified by quartiles (the first quartile, ≤3.6 mg/dL; the second quartile, >3.6–5.1 mg/dL; the third quartile, >5.1–6.9 mg/dL; and the fourth quartile, >6.9 mg/dL). The associations between SUA levels and in-hospital mortality of AKI were assessed with multivariable logistic regression and expressed as the adjusted odds ratio (OR) with its associated 95% confidence interval (CI). We used two multivariable models to determine the significant association of SUA levels with in-hospital mortality. We selected potential confounders based on confounding variables with p values <0.05 in the univariate analysis and based on our team’s clinical expertise. In model 1, covariates were adjusted for age, sex and BMI. In model 2, we adjusted for age; sex; BMI; emergency status; AKI stage; the presence of CKD, diabetes, hypertension, heart disease, or cancer; creatinine; ALB; cholesterol; triglyceride; Hb; eGFR and renal replacement therapy (RRT) requirement; and the use of ACEIs, ARBs, beta-blockers, CCBs, furosemide and UA-lowering agents. The variance inflation factor (VIF) method was used to test for multicollinearity, with VIF ≥5 indicating the presence of multicollinearity. Stratified analyses were performed to explore whether the association between SUA levels and in-hospital mortality differed across various subgroups by age, sex, different AKI stages, the use of UA-lowering agents or furosemide, and the presence of CKD or diabetes. We generated receiver operating characteristic (ROC) curves to measure the sensitivity and specificity of SUA levels and age and calculated the area under the curve (AUC) to ascertain the quality of SUA levels as a predictor of mortality.

Two-tailed tests were performed, and *p* < 0.05 was considered indicative of statistical significance. Statistical analyses were performed using Stata 14.0 (Stata Corp., College Station, TX, USA).

## Results

### Baseline characteristics

**Figure 1.**
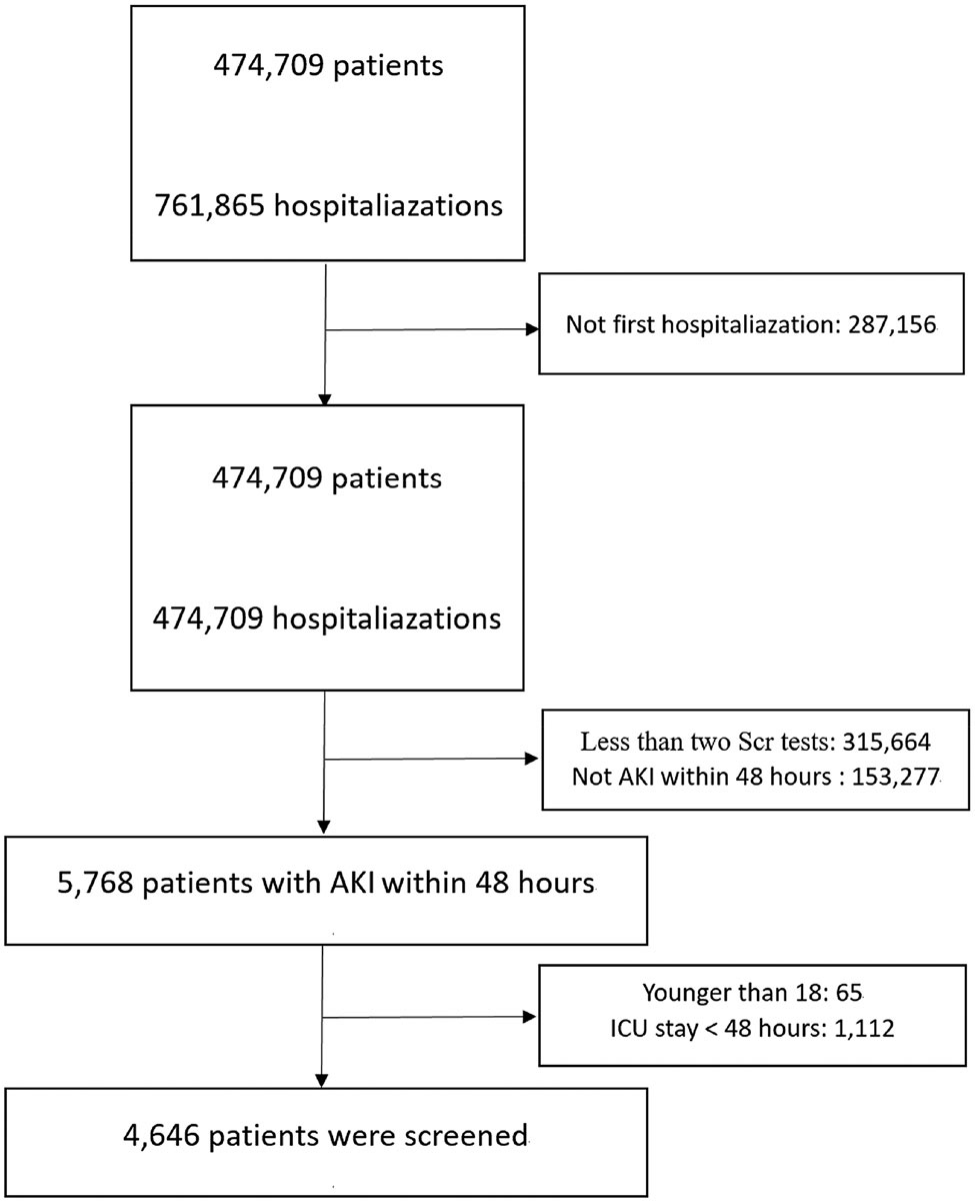
Flow chart of patient selection from the database.

According to the inclusion and exclusion criteria, 4646 patients with AKI within 48 h after hospital admission were included (Figure 1). Among all patients, the mean age was 58.1 years, and 2491 (53.6%) were men. The average SUA level was 5.5 mg/dL for all patients; of whom 23.8% had hyperuricemia and 2.8% used UA-lowering agents. AKI stages 1, 2 and 3 accounted for 89.7%, 6.0% and 4.3% of patients, respectively. Compared with the lowest SUA group (≤3.6 mg/dL), the highest SUA group (>6.9 mg/dL) had a higher proportion of patients with CKD and hypertension.

Regarding laboratory parameters, patients with higher SUA levels also had higher BMI, creatinine and triglyceride values but lower hematocrit and eGFR values. Moreover, they were more likely to require RRT than patients with lower SUA (≤3.6 mg/dL). Other baseline data are shown in Table 1.

### Association between SUA levels and clinical outcomes

**Figure 2.**
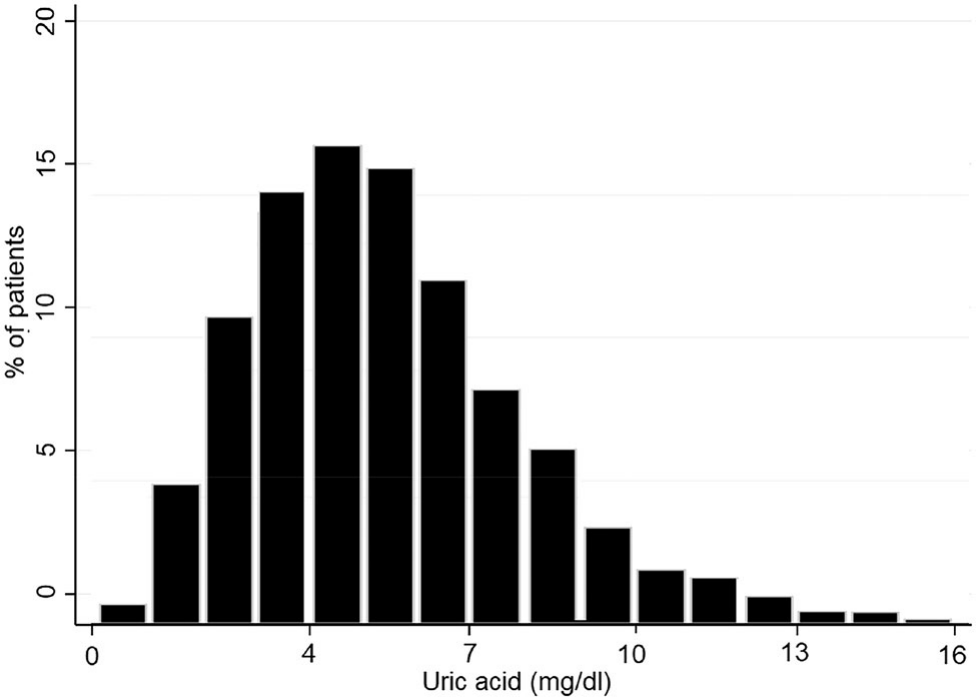
Histogram of serum uric acid distribution.

**Figure 3.**
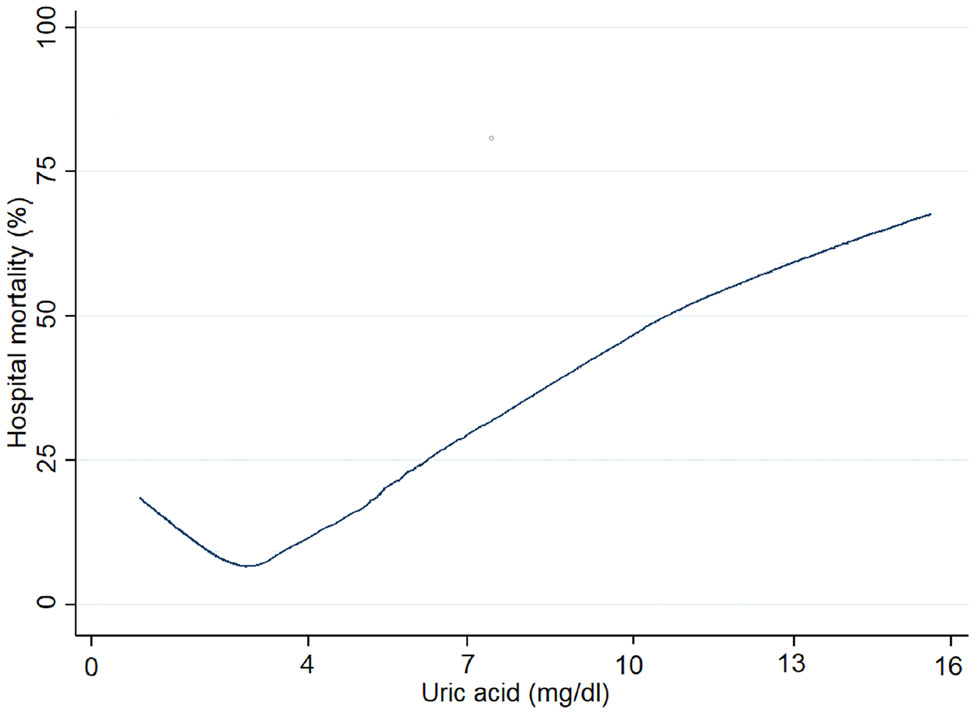
Nonlinear relationship between SUA level and in-hospital mortality in AKI patients.

**Figure 4.**
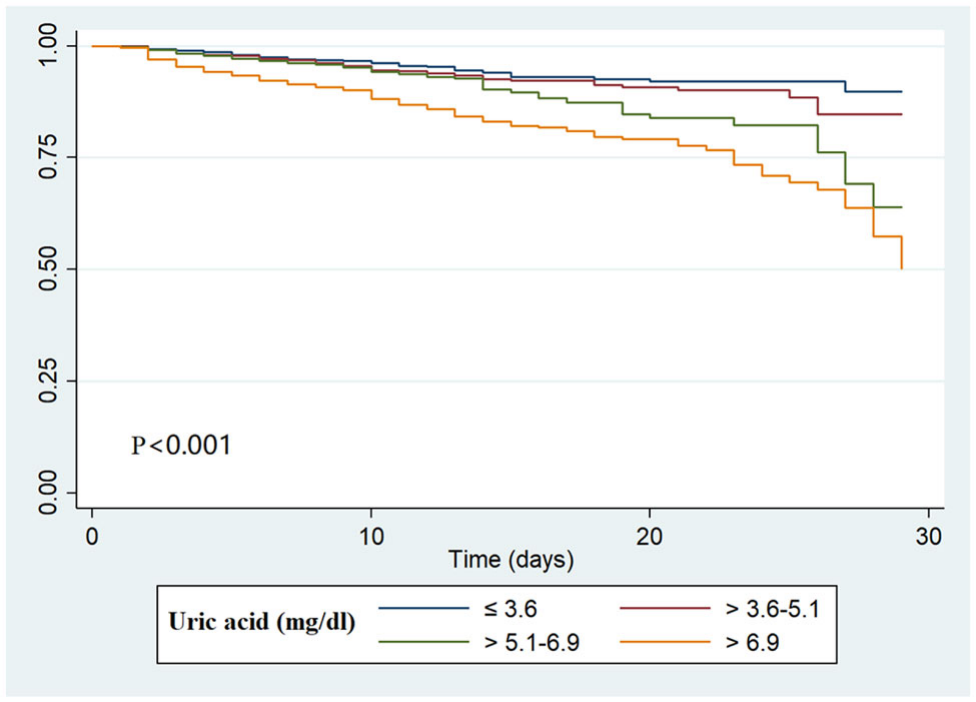
Kaplan–Meier survival curves of the study population.

The distribution histogram of SUA levels is shown in Figure 2. A total of 358 (7.7%) AKI patients died during hospitalization. The results of our unadjusted spline analyses are presented in Figure 3 and showed a J-shaped curve of the association between SUA and in-hospital mortality. Kaplan-Meier survival curves describe the survival distribution of patients grouped according to SUA quartiles within 30 d of admission. Patients in the higher SUA group had worse survival (Figure 4). Subsequently, multivariable logistic regression was used to assess the correlation between SUA levels and the in-hospital mortality of AKI patients (Table 2). In multivariable analysis of model 1, which was adjusted for age, sex and BMI, the ORs were 1.46 (95% CI, 1.01–2.12) in the SUA level >5.0–6.9 mg/dl group and 3.16 (95% CI, 2.25–4.45) in the SUA level >6.9 mg/dl group compared with the reference group (SUA ≤3.6 mg/dl). After adjustment for age; sex; BMI; emergency status; AKI stage; the presence of CKD, diabetes, hypertension, heart disease, or cancer; creatinine; ALB; cholesterol; triglyceride; Hb; eGFR; RRT requirement; and the use of ACEIs, ARBs, beta blockers, CCBs, furosemide and UA-lowering agents, a similar trend was observed in analysis of model 2. A higher SUA level was associated with an increased risk of in-hospital mortality in AKI patients (SUA levels >5.1–6.9 mg/dl versus ≤3.6 mg/dl: OR, 1.72, 95% CI, 1.21–2.33, *p* = 0.005; SUA levels >6.9 mg/dl versus ≤3.6 mg/dl: OR, 2.75, 95% CI, 1.78–4.26, *p* < 0.001).

**Table 2.** Odds ratio for in-hospital mortality according to SUA levels.

SUA (mg/dl)	Unadjusted			Model 1			Model 2		
	OR	95% CI	*p* Value	OR	95% CI	*p* Value	OR	95% CI	*p* Value
≤3.6	reference			reference			reference		
>3.6–5.1	1.37	0.94–2.01	0.098	1.31	0.89–1.92	0.172	1.24	0.82–1.88	0.306
>5.1–6.9	1.67	1.16–2.39	0.006	1.46	1.01–2.12	0.044	1.72	1.21–2.33	0.005
>6.9	3.64	2.62–5.05	<0.001	3.16	2.25–4.45	<0.001	2.75	1.78–4.26	<0.001

CI: confidence interval; OR: odds ratio; SUA: serum uric acid; AKI: acute kidney injury; CKD: chronic kidney disease; RRT: renal replacement therapy; eGFR: estimated glomerular filtration rate; BMI: body mass index.

Logistic regression: model 1 was adjusted for age, sex and BMI; model 2 was adjusted for age; sex; BMI; emergency status; AKI stage; presence of CKD, diabetes, hypertension, heart disease, or cancer; creatinine; albumin; cholesterol; triglyceride; Hb; eGFR and RRT requirement; and the use of angiotensin-converting enzyme inhibitors, angiotensin receptor blockers, beta blockers, calcium channel blockers, furosemide and UA-lowering agents.

The mean variance inflation factor was 1.03 and 1.90 for Model 1 and Model 2, respectively.

Moreover, multivariable logistic regression was used to estimate the association between SUA levels and the rate of nonrecovery of renal function among AKI patients (Table 3). In the multivariable analysis of model 1, after adjustment for age, sex and BMI, the OR for AKI patients with SUA levels >6.9 mg/dl whose renal function had not recovered was higher than that in those with SUA levels ≤3.6 mg/dl (OR, 1.59, 95% CI, 1.07–1.98, *p* < 0.001). A similar trend was observed for model 2 after adjustment for various confounding factors (SUA levels >6.9 mg/dl versus ≤3.6 mg/dl: OR, 1.46, 95% CI, 1.25–1.85, *p* < 0.001).

**Table 3.** Odds ratio for nonrecovery of AKI according to SUA levels.

SUA (mg/dl)	Unadjusted			Model 1			Model 2		
	OR	95% CI	*p* Value	OR	95% CI	*p* Value	OR	95% CI	*p* Value
≤3.6	reference			reference			reference		
>3.6–5.1	1.03	0.88–1.22	0.671	1.09	0.92–1.29	0.340	1.14	0.86–1.52	0.167
>5.1–6.9	1.15	0.88–1.33	0.120	1.12	0.79–1.30	0.428	1.07	0.92–1.20	0.258
>6.9	1.51	1.12–2.01	0.012	1.59	1.07–1.98	<0.001	1.46	1.25–1.85	<0.001

CI: confidence interval; OR: odds ratio; SUA: serum uric acid; AKI: acute kidney injury; CKD: chronic kidney disease; RRT: renal replacement therapy; eGFR: estimated glomerular filtration rate; BMI: body mass index.

Logistic regression: model 1 was adjusted for age, sex and BMI; model 2 was adjusted for age; sex; BMI; emergency status; AKI stage; presence of CKD, diabetes, hypertension, heart disease, or cancer; creatinine; albumin; cholesterol; triglyceride; Hb; eGFR and RRT requirement; and the use of angiotensin-converting enzyme inhibitors, angiotensin receptor blockers, beta blockers, calcium channel blockers, furosemide and UA-lowering agents.

The mean variance inflation factor was 1.03 and 1.90 for Model 1 and Model 2, respectively.

### Subgroup analysis

We analyzed the association between SUA levels and in-hospital mortality in different subgroups by age, sex, AKI stage, the use of UA-lowering agents and furosemide, and the presence of CKD and diabetes (Table 4). Significant interactions were observed for age, sex, AKI stage, presence of diabetes and the use of UA-lowering agents (*p* < 0.001). Patients with a high SUA level who were male (OR 1.14, 95% CI, 1.07–1.21) and younger than 65 years (OR 1.11, 95% CI, 1.03–1.20) were more likely to survive. A similar trend was observed for patients with stage 1 AKI (OR 1.20, 95% CI, 1.14–1.27), those without diabetes (OR 1.17, 95% CI, 1.10–1.24), UA-lowering agent use (OR: 1.16, 95% CI, 0.97–1.38), and furosemide use (OR: 1.18, 95% CI, 1.11–1.25).

**Table 4.** Subgroup analyses of the association between SUA and in-hospital mortality.

Variables	N	OR (95% CI)	*p* value	*p* for interaction
Age				<0.001
<65	2771	1.11 (1.03–1.20)	0.007	
≥65	1875	1.20 (1.12–1.28)	<0.001	
Sex				<0.001
Male	2491	1.14 (1.07–1.21)	<0.001	
Female	2155	1.27 (1.16–1.39)	<0.001	
AKI stage				<0.001
Stage 1	4169	1.20 (1.14–1.27)	<0.001	
Stage 2	279	1.52 (1.20–1.71)	<0.001	
Stage 3	198	1.61 (1.31–1.97)	<0.001	
UA-lowering agents				<0.001
No	4513	1.19 (1.13–1.25)	<0.001	
Yes	133	1.16 (0.97–1.38)	0.109	
Furosemide				<0.001
No	3508	1.23 (1.18–1.28)	<0.001	
Yes	1138	1.18 (1.11–1.25)	<0.001	
CKD				0.686
No	3837	1.16 (1.10–1.22)	<0.001	
Yes	809	1.27 (1.11–1.46)	<0.001	
Diabetes				<0.001
No	3495	1.17 (1.10–1.24)	<0.001	
Yes	1151	1.31 (1.18–1.45)	<0.001	

CI: confidence interval; OR: odds ratio; UA: uric acid; AKI: acute kidney injury; CKD: chronic kidney injury.

### Prediction of in-hospital mortality for AKI

**Figure 5.**
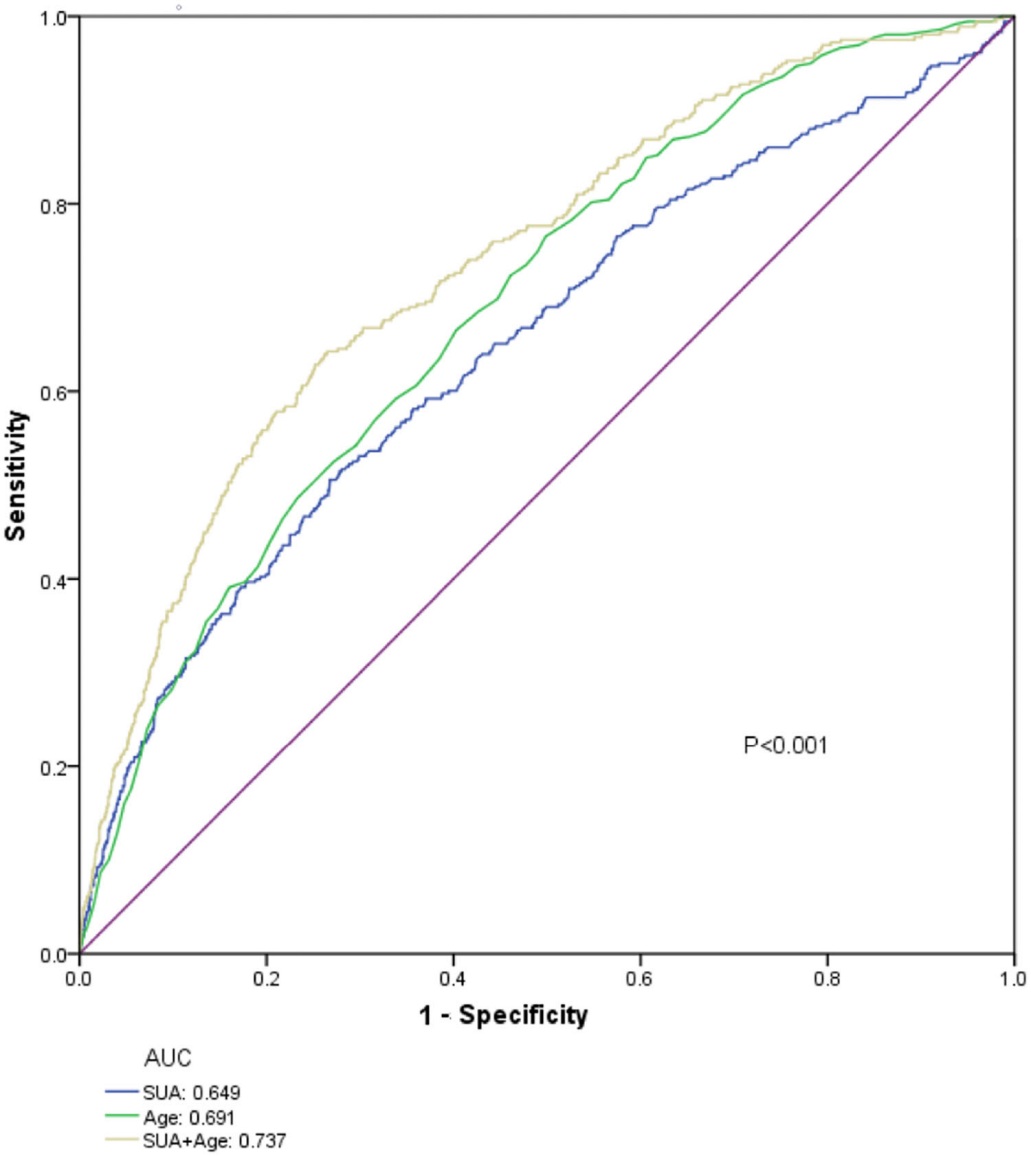
ROC curves for the prediction of mortality in patients with AKI.

ROC analysis was applied to assess the predictive ability of SUA levels for in-hospital mortality in patients with AKI. ROC curves generated using the indicated variables (age, SUA level, SUA level combined with age) are plotted in Figure 5. In ROC analysis for mortality prediction, with the AUC of age (0.691) as the reference, the AUC of SUA level was 0.65 with a sensitivity of 51% and a specificity of 73% (Table 5). Furthermore, when SUA level was combined with age to predict in-hospital mortality in AKI patients, the AUC reached the maximum value of 0.74; in addition, the sensitivity and specificity increased to 64% and 74%, respectively (Table 5).

**Table 5.** Performance of SUA levels for predicting in-hospital mortality in patients with AKI.

Variables	AUC (95% CI)	Sensitivity	Specificity	*p* Value
SUA	0.65 (0.62–0.68)	0.51	0.73	<0.001
Age	0.69 (0.66–0.72)	0.67	0.60	<0.001
SUA + Age	0.74 (0.71–0.77)	0.64	0.74	<0.001

## Discussion

In this retrospective analysis, our results showed a J-shaped curve of the association between SUA levels and in-hospital mortality of patients with AKI within 48 h after hospital admission, and elevated SUA levels were associated with increased in-hospital mortality and rate of nonrecovery of renal function in those patients. ROC analysis demonstrated that SUA level may be an independent prognostic marker for these patients.

AKI is the most common life-threatening complication in hospitalized patients and has a high mortality rate [26]. Previous studies have demonstrated that systemic and local inflammation are closely associated with the occurrence and development of AKI [27]. Several studies have been proposed to investigate the mechanisms underlying the systemic inflammatory response in patients with AKI. Moreover, iron metabolism and bone marrow function may be inhibited by the systemic inflammatory response [28]. The release of proinflammatory cytokines inhibits erythrocyte maturation and proliferation [29]. In addition, the degree of inflammation has a considerably negative impact on patient survival [30]. Many studies have confirmed a certain correlation between high SUA levels and the inflammatory response, oxidative stress and activation of the renin-angiotensin system [31]. Thus, systemic inflammatory responses may help explain the potential association between high SUA levels and mortality in AKI patients. Moreover, fundamental changes in renal vascular and vasoconstriction mechanisms that occur in AKI are similar to those in hyperuricemia, including renin-angiotensin-aldosterone system (RAAS) activation, oxidative stress, nitric oxide reduction, and inflammation [31]. These findings revealed a strong association between high SUA levels and AKI. However, the exact mechanism of these relationships remains unknown.

A number of *in vitro* and *in vivo* studies have supported that hyperuricemia is a risk factor for renal and cardiovascular diseases and leads to inflammation, oxidative stress, endothelial dysfunction and RAAS activation [14]. However, UA, an essential component in the body, plays two main roles [32]. First, UA, along with ammonia and urea, is important for the elimination of nitrogenous compounds [33]. Second, UA is a potent antioxidant, providing more than half of the free‐radical scavenging capacity in the serum of healthy people [34]. Its antioxidant mechanism is related to the protection of the microvascular endothelium, including the prevention of oxidative inactivation of endothelial enzymes and preservation of the endothelial mediation of vascular dilatation under conditions of oxidative stress [34]. On the other hand, UA can form stable complexes with iron ions, which can dramatically inhibit Fe3+‐catalyzed ascorbate oxidation and lipid peroxidation [35]. Therefore, a certain concentration of UA in the body has a protective role. Our study shows a J-shaped curve of the association between SUA levels and in-hospital mortality in patients with AKI within 48 h after hospital admission. This suggests that in-hospital mortality is the lowest when SUA levels are in the appropriate range. However, when SUA levels are higher or lower than the appropriate range, in-hospital mortality will increase. This conclusion is consistent with that of a study on patients with CKD [14]. Therefore, it may be reasonable to monitor SUA concentrations more actively for AKI patients with hyperuricemia and in a timely manner to maintain SUA concentrations in an appropriate range.

The kidneys regulate circulating UA levels by excreting approximately 70% of the UA and reabsorbing approximately 90%∼95% of the filtered urate in the proximal renal tubules [8]. Therefore, SUA levels are associated with glomerular filtration and tubule reabsorption. Causes of high SUA levels may include reduced eGFR, decreased renal tubule UA secretion, enhanced renal tubule reabsorption capacity, or excessive UA production caused by metabolic diseases [36]. AKI is usually accompanied by a decrease in eGFR or injury of the renal tubules and renal interstitium, which may be associated with an increase in SUA levels in AKI patients. Among the AKI stage subgroups, we found that SUA levels were significantly associated with higher mortality in all stages of AKI and that this association was more pronounced in the higher AKI stages.

There is growing evidence that AKI patients with CKD have a different prognosis than those without CKD. Ali et al. reported that the in-hospital mortality of AKI patients with CKD was significantly higher than that of AKI patients without CKD [37]. Wu et al. found that the renal recovery rate of AKI patients without CKD was significantly higher than that of AKI patients with CKD [38]. High SUA levels are closely associated with CKD and are considered not only an important indicator of renal insufficiency but also an important factor in the progression of CKD. However, in our subgroup study, we did not find a significant interaction between SUA levels and in-hospital mortality in patients with or without CKD. This may be related to differences in the study population.

The key clinical question is whether UA-lowering therapy can reduce the mortality risk in patients with AKI. Some studies have shown that the application of allopurinol can reduce SUA levels and improve the systemic inflammatory response and oxidative stress [39]. In a rat model of cisplatin-induced AKI, hyperuricemic rats treated with UA-lowering agents showed significant reductions in intrarenal inflammation and improvements in renal tubular injury compared with hyperuricemic rats not treated with these agents [40]. A prospective clinical study confirmed that among patients with hyperuricemia undergoing high-risk heart surgery, the uric acid oxidase-treated group should have less renal structural damage than the placebo-treated group [41]. Another study confirmed that UA oxidase application decreased the level of serum uric acid and increased the mean eGFR from 55 mL/min/1.73 m^2^ to 136 mL/min/1.73 m^2^ in the treatment of tumor lysis syndrome in children with advanced mature B-cell non-Hodgkin lymphoma [42]. In this study, we found that there was a significant interaction between SUA levels and in-hospital mortality in patients who were or were not treated with UA-lowering agents. This finding indicated that the use of UA-lowering agents may be beneficial for patients with AKI. However, the effect of UA-lowering agents on high SUA levels in AKI patients should be confirmed by prospective randomized controlled trials.

This study has several potential limitations. First, due to the difficulty in obtaining urine values, this study used only creatinine criteria to define AKI, and the sensitivity and specificity of AKI may be different from those in previous studies. Second, in our study, we did not analyze long-term mortality because we did not have access to mortality rates after discharge. Third, we excluded some patients who were missing serum creatinine or serum uric acid data or who had other incomplete data that might be affected by selection bias. Fourth, this is a retrospective study. Despite the correction for confounders, residual confounding cannot be completely excluded. Therefore, prospective studies are needed to confirm our conclusions. Finally, this was a single-center study, and our conclusions need to be further verified by multicenter trials.

## Conclusions

We found that the SUA concentration appeared to be an independent prognostic marker of in-hospital mortality in AKI patients within 48 h after hospital admission and that elevated SUA levels were associated with an increased risk of in-hospital mortality and rate of nonrecovery of renal function in these patients.

## Supplementary Material

Supplemental Material
