# Supplementary material for: Association between serum uric acid levels and clinical outcomes in patients with acute kidney injury
Source: Ren Fail. 2023 Apr 19;45(1):2169617. doi: 10.1080/0886022X.2023.2169617 (PMC10120524; doi:10.1080/0886022X.2023.2169617)
Supplement: Supplemental Material [file IRNF_A_2169617_SM9816.pdf]

Table S1. Number of patients (%) for serum creatinine tests

Number of serum creatinine tests	Number (%)
2	93585(58.8%)
3	30070(18.9%)
4	16164(10.2%)
5	7500(4.7%)
6	4204(2.6%)
7	2108(1.3%)
8	1394(0.9%)
9	778(0.5%)
10	600(0.4%)
>10	2642(1.7%)

The average number of tests for serum creatinine was 3.

Information Classification: General
